# PW03-034 – How to classify autoinflammatory diseases?

**DOI:** 10.1186/1546-0096-11-S1-A260

**Published:** 2013-11-08

**Authors:** G Grateau, V Hentgen, K Stankovic, I Jéru, S Amselem, O Steichen

**Affiliations:** 1 Université Pierre Et Marie Curie Paris 6, Paris, France; 2 Pédiatrie, Centre Hospitalier, Le Chesnay, France; 3 Hôpital Tenon, France; 4 Génétique, INSERM UMR 933, Paris, France

## Introduction

Definitions and classifications of autoinflammatory diseases have been multiple. Their succession highlights the advances in our understanding of the innate immune system, especially the role of interleukin 1β and the inflammasome. However, these definitions and classifications face a number of structure and content issues.

## Objectives

To propose a novel definition of autoinflammatory diseases and to challenge the global classification of inflammatory diseases.

## Methods

We appeal to the desirable characteristics of classification systems (exhaustiveness, disjointness, naturalness, usefulness) and to a critical analysis of the notion of continuum.

## Results

We propose a clinically-oriented definition: “autoinflammatory diseases are diseases with clinical signs of inflammation, associated with elevated acute phase reactants and due to a dysfunction in the innate immune system, genetically determined or triggered by an endogenous factor”.

It is hard to find natural properties able to underlie a useful classification of autoinflammatory diseases, and inflammatory diseases as a whole, into disjoint and exhaustive categories. The notion of continuum is therefore appealing. However, a single continuum from purely autoinflammatory to purely autoimmune diseases oversimplifies, and even distorts, reality. How to locate, for instance, the disease caused by a deletion in PLCG2 (the gene encoding phospholipase Cγ2) that associates autoinflammatory symptoms to both common variable immunodeficiency and autoimmune features? Here we have an overactivation of both the innate and the adaptive immune system, associated with a deficiency of the adaptive immune system.

More than one dimension is needed to properly represent the immunological dysfunctions underlying inflammatory diseases. Furthermore, a classification of inflammatory diseases should also make sense of the clinical, pathological and biological phenotypes.

## Conclusion

To be adequate and useful, a definition of autoinflammatory diseases and a classification of inflammatory diseases must take the multiple facets of reality into account, including clinical features. This can be done within a continuum only if it is multidimensional.

## Disclosure of interest

None declared

